# Identification and evolutionary analysis of novel exons and alternative splicing events using cross-species EST-to-genome comparisons in human, mouse and rat

**DOI:** 10.1186/1471-2105-7-136

**Published:** 2006-03-15

**Authors:** Feng-Chi Chen, Chuang-Jong Chen, Jar-Yi Ho, Trees-Juen Chuang

**Affiliations:** 1 Genomics Research Center, Academia Sinica, Academia Road, Nankang, Taipei 11529, Taiwan

## Abstract

**Background:**

Alternative splicing (AS) is important for evolution and major biological functions in complex organisms. However, the extent of AS in mammals other than human and mouse is largely unknown, making it difficult to study AS evolution in mammals and its biomedical implications.

**Results:**

Here we describe a cross-species EST-to-genome comparison algorithm (ENACE) that can identify novel exons for EST-scanty species and distinguish conserved and lineage-specific exons. The identified exons represent not only novel exons but also evolutionarily meaningful AS events that are not previously annotated. A genome-wide AS analysis in human, mouse and rat using ENACE reveals a total of 758 novel cassette-on exons and 167 novel retained introns that have no EST evidence from the same species. RT-PCR-sequencing experiments validated ~50 ~80% of the tested exons, indicating high presence of exons predicted by ENACE. ENACE is particularly powerful when applied to closely related species. In addition, our analysis shows that the ENACE-identified AS exons tend not to pass the nonsynonymous-to-synonymous substitution ratio test and not to contain protein domain, implying that such exons may be under positive selection or relaxed negative selection. These AS exons may contribute to considerable inter-species functional divergence. Our analysis further indicates that a large number of exons may have been gained or lost during mammalian evolution. Moreover, a functional analysis shows that inter-species divergence of AS events may be substantial in protein carriers and receptor proteins in mammals. These exons may be of interest to studies of AS evolution. The ENACE programs and sequences of the ENACE-identified AS events are available for download.

**Conclusion:**

ENACE can identify potential novel cassette exons and retained introns between closely related species using a comparative approach. It can also provide information regarding lineage- or species-specificity in transcript isoforms, which are important for evolutionary and functional studies.

## Background

Alternative splicing (AS) is suggested to be a major source of transcriptome/proteome complexity and gene function diversity [1-7], and highly relevant to several human diseases [8-10]. AS is also reported to alter important protein features, such as phosphorylation, glycosylation, and transmembrane helices [11]. Bioinformatics studies based on expressed sequence tag (EST) database estimate that between 30% and 60% of all human genes undergo AS [1-3,12,13]. However, the exact extent of AS in the human genome remains uncertain and EST/mRNA information in mammals other than human and mouse is still very limited. Therefore, it is likely that a considerable number of human AS transcripts remain unknown, and even more AS variants are to be discovered in other EST-scanty mammals. Moreover, adequate information of AS variants in more mammalian species other than human and mouse can further our understanding on the evolution of AS and its implications in functional divergence. Hence, it is desirable to identify unannotated AS variants and evolutionarily meaningful AS events in mammals, particularly in species of which AS events have remained largely undiscovered.

AS exons are suggested to evolve fast in mammals [14,15]. The high evolution rate of AS exons may have caused remarkable inter-species divergence of AS patterns, which in turn can result in different protein structures and functions. To further understand mammalian functional divergence in view of AS evolution, it is important to analyze species-specific AS exons (exons that are alternatively spliced in one species but constitutively spliced in others) and conserved AS exons (exons that are alternatively spliced in compared species). Species-specific AS variants can result in functional novelty by changing the length, composition, structure, and/or transcriptional and translational regulation of proteins in the affected species, leading to remarkable functional disparity between orthologous proteins [16]. On the other hand, conserved AS variants represent functionally important transcripts that tend to preserve such features as length, exon-intron boundaries, number of exons, reading frame, introns flanking AS exons, and so on [5,17-21]. In other words, conserved AS variants serve to form the framework of critical biological functions across species, while species-specific AS events constitute a mechanism to develop novel protein functions that occur only in the affected species. Therefore, it is important to perform a systematical analysis to identify potential species-specific and conserved AS events in mammals.

The simplest way of determining species-specificity of AS events is to check inter-species differences in AS patterns based on EST information. However, the limited availability of EST information seriously restricts the application of such approaches. In contrast, comparative computational approaches can circumvent this shortcoming and identify AS events without EST data. Nevertheless, previous comparative computational studies mainly focus on identification of cassette-off exons (exons that were annotated as constitutive, but later found to be alternative) based on sequence features of conserved AS variants [18-24]. Meanwhile, cassette-on exons (exons that were not previously annotated) and retained introns (newly annotated exonic sequences that cover the full length of introns) have remained relatively unexplored. Note that these cassette-on exons and retained introns represent not only novel AS events but also novel exonic sequences, which are different from cassette-off exons because the latter represent novel AS events formed by known exons. These two AS exon types are important because they can insert additional amino acids into existing proteins and potentially can alter the structure and/or function of the affected proteins. In particular, retained introns may cause drastic changes by changing the number of exons and eliminating existing exon-intron boundaries. Therefore, it is desirable to identify these less studied AS exons and infer their functional and evolutionary implications in mammals. With the combination of cassette-on exon, retained intron, and cassette-off exon, a more complete view of AS evolution in mammals can be obtained than if only one of these AS exon types is studied.

In this study, we propose a new cross-species EST-to-genome approach named ENACE (Extracting Novel Alternative splicing variants from Cross-species EST resources), which extracts novel exonic sequences of one species (the "target species") from ESTs of another species (the "source species"). The extracted exons are compared with existing EST libraries to confirm that they do not overlap any ESTs of the target species. As three species (human, mouse, and rat) are considered here, the study consists of six ENACE identification processes: E_H-m_, E_M-h_, E_H-r_, E_R-h_, E_M-r_, and E_R-m_, where the uppercase subscript stands for the target species and the lowercase subscript for the source species. For example, E_H-m _indicates the ENACE process for extracting human (the target species) novel exonic regions based on mouse (the source species) ESTs. We denote the ENACE predicted novel exonic regions as the "ENACE exons". Here "novel" indicates lack of annotations in public databases (e.g., UCSC or NCBI) or supporting evidence from the-same-species ESTs for the existence of ENACE exons. Note that all the ENACE exons are either cassette-on exons or retained introns. For the novel exons identified, it is of primary interest to understand their biological and evolutionary significance. Two functional features of ENACE exons, including potential protein domains and gene ontology (GO) categories, are analyzed. In addition, two evolutionary analyses, namely the nonsynonymous-to-synonymous substitution ratio test (or the *K*_*A*_*/K*_*S *_ratio test [25,26]) and calculation of the conservation level of ENACE exons among human, mouse, and rat, are performed. Since ENACE is a comparative approach, we are able to study species-specific exons or exon gain/loss events that occurred during mammalian evolution by analyzing ENACE results. The evolutionary implications of ENACE results are also discussed.

## Results and discussion

### Outline of the ENACE strategy

**Figure 1:**
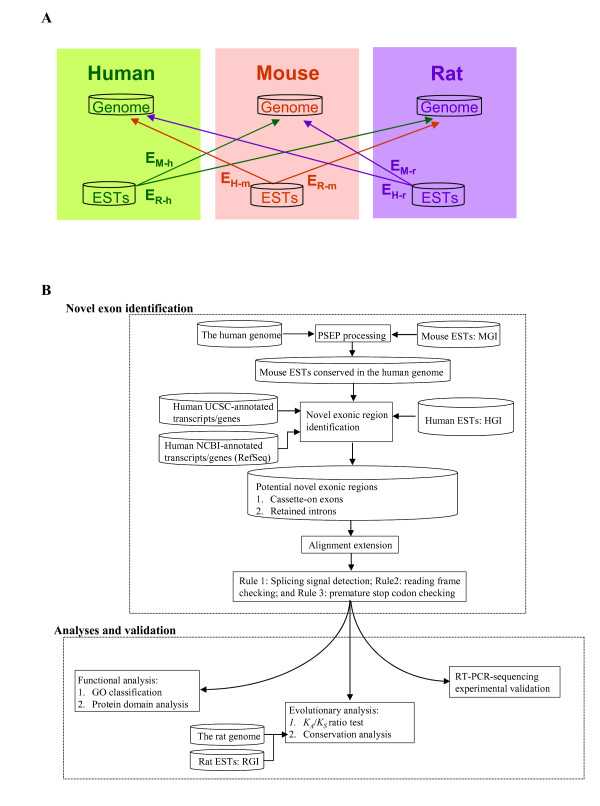
The ENACE analysis process. **(A) **Six ENACE processes: E_H-m_, E_M-h_, E_H-r_, E_R-h_, E_H-r_, E_M-r_, and E_R-m_. Arrows indicate mapping of ESTs from the source species to the genome of the target species. See text for more details. **(B) **An example of ENACE process for E_H-m_. ENACE is implemented with two major components: novel exon identification; and analyses and validation of the identified protein-coding exons.

**Figure 2:**
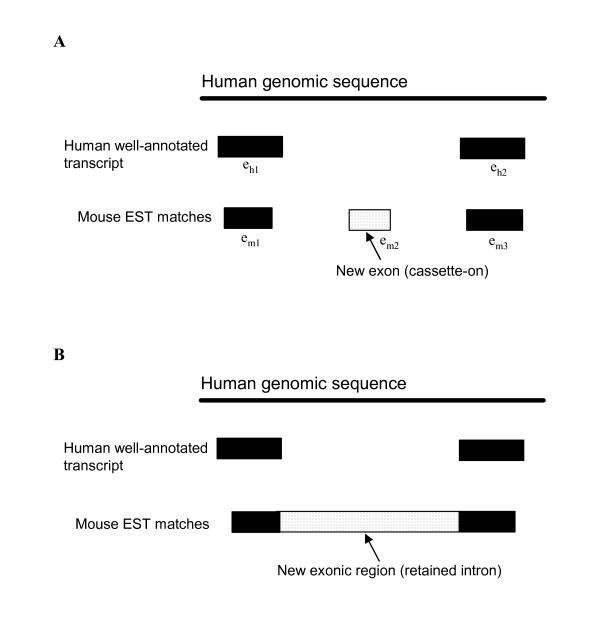
Two types of novel AS exons identified in this study. Shown in the figure is the mapping of a mouse EST to the human genome. **(A) **Cassette-on exon. e_h1 _and e_h2 _are well-annotated human exons. e_m1_, e_m2_, and e_m3 _are segments of a mouse EST sequence, where e_m1 _and e_m3 _respectively overlap with e_h1 _and e_h2_, and e_m2 _matches the human intron between e_h1 _and e_h2_. e_m2 _is therefore identified as a potential novel cassette-on exon of human. **(B) **Retained intron. The matching EST segment is identified as a retained intron, if it covers a whole intron between two adjacent human exons.

ENACE makes use of cross-species EST-to-genome conservation to extract novel exonic sequences in human, mouse, and rat. The novel exonic sequences identified can be also regarded as AS events. Figure 1 illustrates the ENACE design. This study includes six ENACE AS identification processes as stated above (Fig. 1A). Here we take the E_H-m _process as an example to describe the ENACE system. As shown in Figure 1B, the proposed ENACE process consists of two major steps: 1. identification of novel exonic regions; and 2. analyses and validation of the AS variants identified. For the first step, we use the PSEP annotator [27] to identify mouse EST fragments conserved in the human genome (see Methods). ESTs thus obtained are compared with known human transcripts (including the human UCSC- and RefSeq-annotated genes/transcripts) and human ESTs. The matching ESTs that overlap with known human transcripts/ESTs are discarded. Thus, the remaining exonic sequences are defined as novel exons with EST evidence from a non-human species but not from human itself. Note that, see Figure 2A for an example, ENACE rules that the flanking exons (i.e., e_m1 _and e_m3_) of the newly identified exon (i.e., e_m2_) must overlap with a known human transcript (i.e., e_h1 _and e_h2_) to avoid false positive detections. Therefore, the ENACE system can only identify internal exons. In addition, the ENACE-identified novel exons are either cassette-on exons (Fig. 2A) or retained introns (Fig. 2B). The extracted cassette-on exons are further processed because the EST-to-genome matching procedure (i.e. the PSEP annotation process) requires high level of sequence identity [27], which may considerably reduce the alignable lengths and make the annotated cassette-on exons shorter than they should be. Therefore, the mouse ESTs that support ENACE-identified cassette-on exons are BLASTed against the corresponding human introns to obtain the maximal alignable sequences (see Methods). If the extended sequences cover the full range of the corresponding introns, they are annotated as retained introns rather than cassette-on exons.

The potential novel exonic regions have to pass the following rules: for cassette-on exons, they must be flanked by AG-GT/AG-GC legal splicing sites (Rule 1); for both cassette-on exons and retained introns that are located in coding sequences (CDS), they must not disrupt the reading frame (Rule 2) and must contain no premature stop codons (Rule 3). Note that a novel exon is referred to as "located in CDS" (or "ENACE CDS exon") if such an exon is located in an intron flanked by coding exons of the target species. On the other hand, if a novel exon is located between an untranslated region (UTR) and a coding exon, or between two UTRs, it is referred to as "located in UTR" (or "ENACE UTR exon").

Exons that pass the three rules mentioned above are further analyzed for functional and evolutionary features, and experimentally validated using RT-PCR-sequencing (Fig. 1B). The functional analyses include protein domain analysis and GO classification, whereas the evolutionary analyses include the *K*_*A*_*/K*_*S *_ratio test and cross-species conservation analysis that determines, for example, whether the identified E_H-m _exons are conserved in the rat genome or ESTs. The workflow stated above also applies to the other five ENACE processes: E_M-h_, E_H-r_, E_R-h_, E_M-r_, and E_R-m_.

### Novel exonic regions identified by ENACE

The numbers of novel exonic regions identified in the six ENACE processes are shown in Table 1. More than 5,000 novel exonic regions (the "meta-ENACE exons") are extracted by ENACE initially, and 925 of them pass the three filtering rules. Of these 925 ENACE exons, 758 (692 cassette-on exons and 66 retained introns) are located in CDS, and the other 167 (141 cassette-on exons and 26 retained introns) in UTR (related information is available [see Additional files 1 and 2]). Note that the number of identified retained introns is much smaller than that of cassette-on exons (Table 1). The reason may be that intron retention is responsible for only a very low percentage (< 3%) of all AS events [24,28] and it exhibits in less than 5% of all genes [29,30]. On the other hand, it is noteworthy that the majority of extracted retained introns in CDS do not pass Rules 2 and 3. A possible explanation is that a substantial number of retained introns may be derived from aberrant or artefactual EST data [31]. Nevertheless, it is also likely that such retained introns have been preserved in the source species while lost in the target species, or they may be gained by the source species after lineage divergence. Either way, these potential lineage-specific retained introns may be evolutionarily important because they may change the number of exons in the affected genes and may influence expression regulation mechanisms such as nonsense-mediated decay [30]. Meanwhile, the number of ENACE UTR exons is smaller than that of ENACE CDS exons because ENACE identifies only internal exons. Whereas ENACE CDS exons are functionally important by inserting extra amino acids, ENACE UTR exons may be critical to transcriptional and translational regulation [32-34]. Therefore, the large number of novel exons identified in this study may lead to new findings in transcriptomics and proteomics studies on the three species involved.

In summary, as many as ~80% of meta-ENACE exons (~4100 exons), which exist in the source species, are observed to lack canonical splicing signals or have disrupted reading frames or premature stop codons in the target species. These exons can be regarded specific to the source species, which may have evolved fast and play a unique part in evolution by increasing the rate of evolutionary changes [14]. On the other hand, the ENACE exons (925 exons) represent both newly identified exons conserved between the target and source species and novel AS events in the target species. Such novel AS events are either specific to the target species (species-specific AS) or they may be conserved between the target and source species (conserved AS). The two scenarios can be distinguished by using existing tools [19-21] to check whether these exons also undergo AS in the source species.

Our results also indicate that two major factors, namely inter-species divergence and EST coverage level, may determine the number of novel exons identified (both cassette-on exons and retained introns) in the ENACE system. Taking cassette-on exons as an example (Table 1), although the number of EST entries for human (HGI, ~835,000 entries) is larger than that for mouse (MGI, ~780,000 entries), the number of novel exons identified in E_H-m _(116 exons) and E_M-h _(119 exons) are very close. Therefore, it is the divergence between human and mouse (rather than the abundance of EST information) that determines how many novel exons can be identified in this case. Another evidence is found in the comparison of cassette-on exons identified in E_H-r _(29 exons) and E_M-r _(105 exons), in which the same EST data set (RGI) is applied but E_H-r _yields a much smaller number of exons because of the longer divergence time between human and rat than between mouse and rat. On the other hand, an example for the influence of EST coverage level can be seen in the comparison between E_H-r _and E_R-h_. Only 29 novel cassette-on exons are identified in E_H-r_, which is much smaller than that for E_R-h _(87 exons), though the two species involved are the same in the two processes. The observation indicates that the number of ENACE exons is positively related to the number of EST entries available. The same situation is also observed in the different results of E_M-r _(105 exons) and E_R-m _(374 exons). Meanwhile, the large number of 374 novel rat exons identified is noteworthy. It shows the value of this cross-species approach because we can identify a considerable number of novel AS transcripts and exons for a species that has only limited EST information (such as rat) by comparing its genomic sequences with the abundant EST information from a closely related species (e.g. mouse).

### Characteristics of ENACE CDS exons

Table 2 shows the basic properties of ENACE CDS exons. Note that the cassette-on exons are relatively short on average (the average and median lengths are 86 bp and 63 bp, respectively), which is consistent with previous studies [20,21,35], but longer than the original source-target exon matches (44 bp and 33 bp for mean and median, respectively). On the other hand, we observe that the average number of ESTs that support the ENACE cassette-on exons is very close to one (1.3), implying that these exons are likely to be minor-form in terms of inclusion level (defined by Modrek and Lee [14]) not only in the target species but also in the source species. The low number of average supporting ESTs for ENACE cassette-on exons does not result from poor EST coverage because comparisons based on human ESTs (E_M-h _and E_R-h_) also yield similarly low numbers of supporting ESTs (1.6 and 1.5, respectively). It has been suggested that more than 80% of minor-form exons fail the *K*_*A*_*/K*_*S *_ratio test [36]. Therefore, most ENACE exons may not be detected by the *K*_*A*_*/K*_*S *_ratio test [25,26] (discussed in a subsequent section). Moreover, the average sequence identity of ENACE exons with their orthologous sequence counterpart is 89% (Table 2), which is lower than the nucleotide identity threshold (at least 95%) between orthologous exon pairs used in some computational approaches [20,21]. Therefore, such computational approaches may not appropriately detect ENACE exons. In short, ENACE provides a unique tool to identify potential novel exons that cannot be detected by other approaches. Although it is likely that predicted transcripts supported by a small number of ESTs are splicing errors, ENACE exons have passed three biological rules that can significantly reduce such possibility. In addition, the supporting ESTs for ENACE exons must include at least three exons, of which the exons flanking the predicted novel exon must be conserved in a known gene of the target species. Therefore, most splicing errors should have been screened out by ENACE processes.

For protein-coding retained introns, it is noteworthy that the average length of the "new" exons that include ENACE retained intron and the flanking exons previously annotated is fairly large (766 bp, see Table 2). The reason is that approximately half of these retained introns are located either between the first and the second coding exon, or between the last and the second last coding exons. The first and the last coding exons are often part of a large exon that also contains UTRs. Therefore, a newly identified retained intron serves to connect such a UTR-including exon and their neighboring coding exon, resulting in a single large exon. It is also worth noting that six of the ENACE retained introns connect the exons of originally two-exon transcripts to make single-exon transcripts (Fig. 2B). Moreover, ENACE retained introns (average length is 115 bp) are significantly shorter than nonretained introns (average length of human introns is about 3.3 kbp [37]), which is consistent with previous observations [30,38].

### Functional analyses of ENACE CDS exons

**Figure 3:**
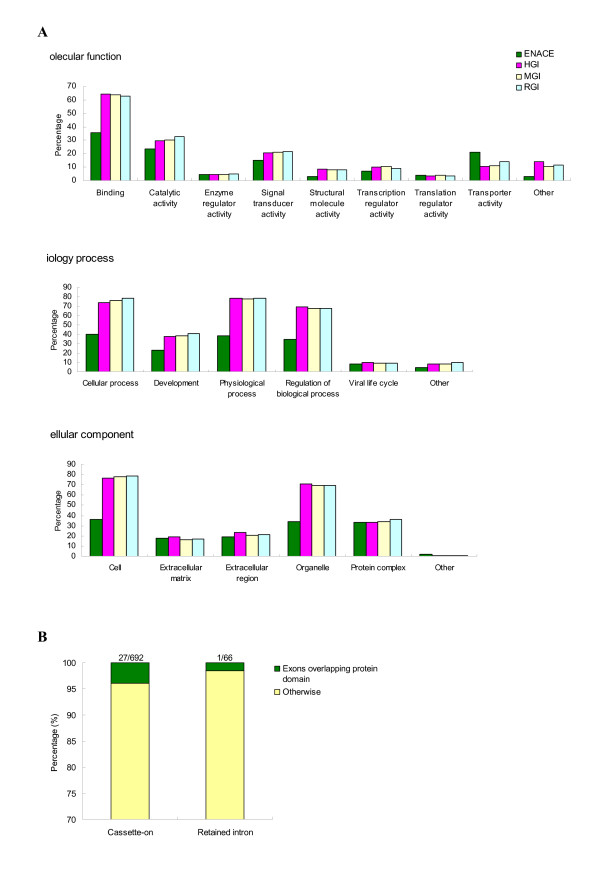
Functional analyses of protein-coding ENACE exons. **(A) **Distribution of ENACE exons and ESTs of Human Gene Index (HGI), Mouse Gene Index (MGI) and Rat Gene Index (RGI) over gene ontology subcategories. **(B) **Proportions of ENACE exons that overlap protein domains.

Figure 3 illustrates the classification of genes that include ENACE CDS exons (the "ENACE genes") according to the three main GO categories (i.e., "molecular function", "biology process", and "cellular component") and the percentage of ENACE exons that overlap protein domains. The GO annotations of HGI, MGI and RGI transcripts are downloaded from the TIGR database. Figure 3A shows that HGI, MGI, and RGI transcripts have similar GO subcategory distributions. Overall, ENACE genes have similar distribution patterns to those of HGI, MGI, and RGI transcripts. However, lower percentages of ENACE genes are assigned GO subcategories than HGI, MGI and RGI because ~50% of ENACE genes are not annotated in the GO database, indicating that about half of the ENACE genes are currently unknown in function. In addition, it appears that the percentages of ENACE genes in each GO subcategory (X-axis) reflect the EST coverage levels of HGI, MGI and RGI except in the "transporter activity" subcategory in "molecular function" (Fig. 3A). Approximately 80% of the ENACE exons in the "transporter activity" subcategory are associated with "protein carrier activity" (~60%) or "receptor protein activity" (~20%). The relatively high percentage of ENACE exons in these subcategories indicate that either a considerable number of protein carrier and receptor protein AS transcripts have not been discovered, or inter-species AS divergence is particularly significant for these protein groups.

For protein domain preservation, as shown in Figure 3B, the majority of ENACE exons do not overlap any protein domains according to the INTERPRO annotation [39]. The finding is consistent with the results of Kriventseva *et al*. [15] and Yeo *et al*[21], who observed that AS tended not to locate within protein domains. This observation implies that the addition of ENACE exons to the affected transcripts may not alter the functional domains in the original protein architecture. Therefore, the normal functions of the affected proteins can be preserved and at the same time the newly added sequences may be allowed to evolve and develop new functions.

### Evolutionary analyses of ENACE CDS exons

To probe the evolutionary significances of the ENACE CDS exons, two analyses are performed: the *K*_*A*_*/K*_*S *_ratio test and conservation of ENACE exons in the ESTs or genome of "the third species" (the species other than the target and source species in this study).

**Figure 4:**
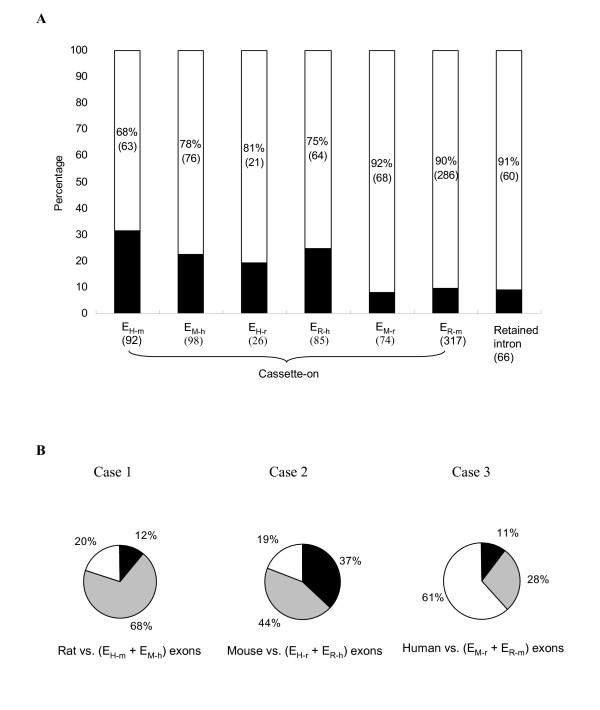
Evolutionary analyses of protein-coding ENACE exons. **(A) **Proportions of ENACE exons that do not pass the *K*_*A*_*/K*_*S *_ratio test (white area) and those that do (black area). **(B) **Comparison of conservation of ENACE exons in the third species other than the target and source species. Case 1: conservation of E_H-m _and E_M-h _exons in rat; Case 2: conservation of E_H-r _and E_R-h_exons in mouse; Case 3: conservation of E_M-r _and E_R-m _exons in human. Black and grey color indicates proportion of ENACE exons conserved in the ESTs and in the genome (but not in the ESTs) of the third species, respectively. White color represents proportion of ENACE exons that are not conserved in the third species.

In Figure 4A, it is clear that the majority of ENACE exons (> ~70%) do not pass the *K*_*A*_*/K*_*S *_ratio test (*K*_*A*_*/K*_*S *_< 1 at the 5% significance level, see Methods) in all ENACE processes. The observation indicates that most of ENACE exons are not subject to stringent selective constraint. The proportion of the ENACE exons that fail the *K*_*A*_*/K*_*S *_ratio test is much larger than that of overall human-mouse orthologous exons (only 9.5%) observed by Nekrutenko et al. [25] The difference can be ascribed to two reasons. First, ENACE exons are likely minor-form exons both in the target and the source species. Such exons are suggested to have higher rates of evolution and may be under relaxed negative selection or positive selection pressure [14]. Second, the lengths of ENACE exons tend to be short. The short lengths of ENACE exons may provide insufficient information for the *K*_*A*_*/K*_*S *_ratio test to be effective [25]. In addition, it is worth noting that the percentages of ENACE cassette-on exons that fail the test in E_M-r _and E_R-m _(≥ 90%) are considerably larger than those in the other four ENACE processes. The reason may be that rodents have a higher mutation rate than human because of their shorter generation time [40,41]. Furthermore, the percentage of ENACE retained introns that fail the test is also larger than 90%. This is because 54 of the 66 retained introns are identified from mouse-rat comparisons. In brief, though the *K*_*A*_*/K*_*S *_ratio test is very powerful in detecting evolutionarily conserved regions, it is not suitable for identifying short and fast-evolving exonic regions such as those identified by ENACE.

For the three-species conservation study, we analyze three cases: Case 1. rat vs. E_H-m _and E_M-h _exons; Case 2. mouse vs. E_H-r _and E_R-h _exons; and Case 3. human vs. E_M-r _and E_R-m _exons (Fig. 4B). For each case, ENACE exons are assigned to one of three conditions: (A) the exons are conserved in both the ESTs and the genome of third species, or (B) they are conserved only in the genome of the third species; or (C) they are not conserved in the third species. Note that an ENACE exon is assigned to condition (B) only when the exon and its flanking exons are all conserved in the third species. This criterion can reduce the possibility of non-specific matching between an ENACE exon and the genome of the third species. Also note that the ENACE exons in condition (C) can be regarded as exons specific to the target and source species (data are available [see Additional file 3]). For example, as shown in Figure 4B, the proportion of the mouse-rat specific ENACE exons (61%) is significantly larger than those of human-mouse and human-rat (~20% for both) specific ENACE exons (*P *< 10^-22 ^and *P *< 10^-15 ^by Fisher's exact test). The reason is clear: mouse and rat are more closely related to each other than they are to human. It is noteworthy that the ~20% of human-mouse (or human-rat) specific exons probably had existed in the common ancestor of human and rodents but were lost in rat (or mouse) after the divergence between mouse and rat. Meanwhile, the 61% rodent-specific exons could be either gained or lost in the rodent lineage after the divergence between human and rodents. In either case, these lineage-specific exons may be evolutionarily important in conveying inter-lineage protein structural or functional divergence.

Also worth noting is the observation that the proportion of condition (A) ENACE exons in Case 2 (37%) is significantly larger than those in both Cases 1 and 3 (*P *< 10^-6 ^and *P *< 10^-9 ^by Fisher's exact test). The reason of the difference between Cases 1 and 2 may be the larger EST coverage in mouse than in rat. Hence a large portion of human-rat ENACE exons can find matches in mouse ESTs, while only a relatively small percentage of human-mouse ENACE exons can find matches in rat ESTs. On the other hand, the difference between Cases 2 and Case 3 may be ascribed partly to the longer divergence time between human and rodents than that between rodents. Overall, our results show that ENACE is capable of identifying not only novel exons but also lineage-specific exons that are involved in AS events, which makes ENACE a convenient tool for AS and evolutionary studies.

### Experimental validation of ENACE CDS exons

**Figure 5:**
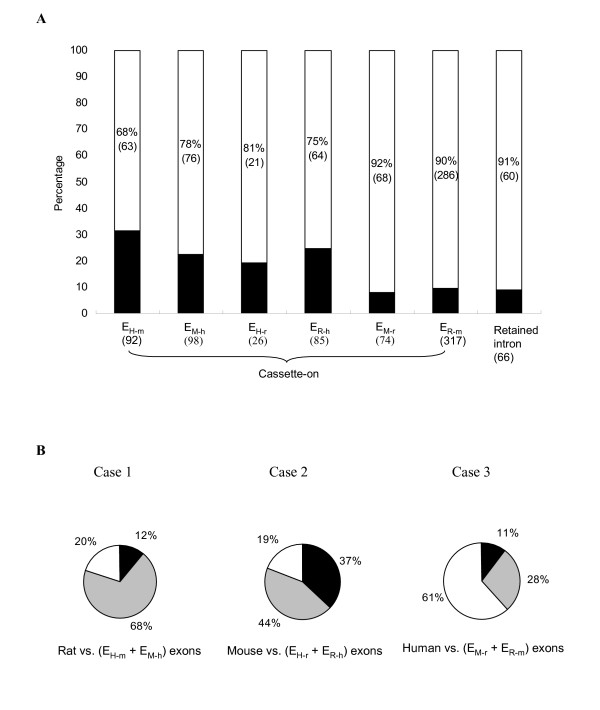
An example of experimental results for novel AS transcript identified by the E_H-m _process. Shown in the figure are RT-PCT products of the human KIAA1109 protein (XP_371706) and its mouse homologue. The 225-bp and 426-bp band represents the well-documented transcript and the novel AS transcript, respectively. The novel AS transcript is expressed in 2/10 human cell lines tested, and in 3/4 mouse cell lines in this study.

To experimentally test the predicted exons, nine sets of ENACE CDS exons are chosen for RT-PCR-sequencing validation. These include cassette-on exons from six ENACE processes, retained introns from human-rodent comparisons (E_H-m_+E_M-h_+E_H-r_+E_R-h_), and retained introns in E_M-r _and E_R-m _(Table 3). An example of RT-PCR results is given in Figure 5, and the validation results are summarized in Table 3. Eighty-three percent of ENACE cassette-on exons from human-mouse comparisons are experimentally validated. Similarly, 50%, 67%, 75%, and 58% for E_H-r_, E_R-h_, E_M-r_, and E_R-m_, respectively, are experimentally verified. For retained introns, 80% for both human-rodent comparisons and E_M-r_, and 60% for E_R-m _are validated. Overall, the experiments indicate that at least 50% of the ENACE exons tested are observed to be novel exons as well as novel alternatively spliced exons in tissues of the target species. These experimental results support the high rates of presence of ENACE exons (complete results are available [see Additional files 4 and 5]).

## Conclusion

In this study we identify as many as ~900 novel exons with a comparative algorithm based on cross-species EST-to-genome comparisons. These novel exons also represent novel AS events because they are either cassette-on exons or retained introns with no previous transcript evidence. Subsequent experimental validation shows that more than 50% of the predicted novel exons are actually included in the transcripts of the target species. The algorithm is different from existing ones because it can identify exons that will otherwise be omitted in other approaches such as the ones based on the-same-species EST information [42-45], computational properties [20,21], or evolutionary properties (e.g. the *K*_*A*_/*K*_*S *_ratio test [25,26,46]). In addition, the cross-species approach has a unique advantage that it can detect AS events and novel exons for EST-scanty species by applying rich EST data from a closely related species. The mouse-rat comparisons exemplify how mouse ESTs can effectively help identify AS exons in rat. Furthermore, the ENACE algorithm is capable of identifying species-specific, lineage-specific and conserved exons, and exons that may be gained or lost in one of the compared species. These exons may be interesting targets for evolutionary and biomedical studies.

## Methods

### Cross-species EST-to-genome comparison by PSEP

We applied PSEP package [27], which is a cross-species gene identification and AS recognition system, to extract cross-species conserved sequences and AS variants. The PSEP system was implemented in two steps: CRASA-based sequence alignments [47] for EST-to-genome/genome-to-genome alignments and a series of progressive signal extracting and patching for exonic region curation. For the first step, PSEP aligned genome of the target species (e.g., human) against ESTs from the target species (e.g., HGI, Human Gene Index), ESTs from the source species (e.g., MGI, Mouse Gene Index), and genome of the source species (e.g., mouse) simultaneously using the CRASA aligner. After completing sequence alignment, post-alignment processes were used to extract exonic regions from the cross-species EST-to-genome alignments. The post-alignment processes included three main steps: reduction of EST-matching results, gap-patching, and analysis of AS transcripts. For reduction of EST-matching results, a large amount of overprediction was filtered out with the aid of conserved sequences identified in the cross-species genome-to-genome and the EST-to-genome alignments. For gap-patching, several rules were applied to progressively deal with gaps and mismatches. Gaps may be patched with reference to high-quality genomic sequence or the EST hits from cross-species EST-to-genome comparisons. For the AS analysis, the patched EST matches, if judged to be redundant, were eliminated by a redundancy-removing rule. Then some filters were applied to further screen out potential artifacts (see Ref. [27] for the detail). The system took account of conserved sequences between the human genome and non-human (e.g., mouse and rat) ESTs, in addition to genome comparison between the two species. With its dual functions in cross-species conserved sequence analysis and AS analysis, PSEP was used here for extracting potentially novel AS patterns including cassette-on exons and retained introns from cross-species ESTs.

### Extension of aligned sequences and screening of extracted cassette-on exons

In the ENACE system, we use the PSEP annotator [27] to perform EST-to-genome comparisons. Since the PSEP algorithm requires high level of identity between compared sequences, the lengths of extracted cassette-on exons can be underestimated. Therefore, we try to extend the lengths of these exons by BLAST-aligning the supporting ESTs against the corresponding introns of the target species. By doing so, we can obtain maximal alignable regions between source ESTs and the target genome. After that, the exon/intron boundaries of the newly annotated exons are also identified, followed by checking the presence of reading frame openness and premature stop-codon. We use the E_H-m _process as an example to describe our analysis scheme. Given a new PSEP-extracted exon e_m2_with its flanking exons e_m1 _and e_m3 _(Fig. 2A), e_h1 _and e_h2 _are well-annotated human exons that overlap with e_m1 _and e_m3 _but not e_m2_. We extracted the human intronic sequence between e_h1 _and e_h2_, and BLAST-aligned this sequence against the corresponding mouse EST sequence located between e_m1 _and e_m3 _to obtain the maximal alignable region. Note that it is possible that the extended region may finally cover the full length of the intron between e_h1 _and e_h2 _and become a retained intron (Fig. 2B). The maximal alignable regions that are not flanked by GT-AG or GC-AG canonical splicing signals are not considered. Finally, the remaining exonic regions with legal exon/intron boundaries are further checked to filter out the ones with frame shifts and premature stop codons. Note that the BLAST package is used with default parameters throughout this study.

### Prediction of protein domains

We detected protein domain overlapping of ENACE exons using the InterProScan package and the INTERPRO resource [39,48-50]. ENACE exons and their flanking exonic sequences were concatenated for InterPro domain scanning.

### *K*_*A*_*/K*_*S *_ratio test

We performed the *K*_*A*_*/K*_*S *_ratio analysis of orthologous exon pairs using the following procedures: (i) calculating the numbers of synonymous and non-synonymous sites, *K*_*A*_, *K*_*S*_, and *K*_*A*_*/K*_*S *_values, using the PAML package [51,52]; (ii) creating two-way contingency tables with rows comprising numbers of synonymous and non-synonymous sites and columns comprising numbers of changed and unchanged sites; and (iii) testing the independence between the numbers of changed synonymous and non-synonymous sites using Fisher's exact test.

### Experimental validation

Human cDNA was obtained by RT-PCR from the following 31 cell lines of 11 different tissue types: stomach (AZ521), liver (HepG2, HepG2/C3A), muscle (TE671), cervical (HeLa), lung (H1299, H460, A549), epithelium (A431, HaCaT), connective tissue (HS-5), embryo (293T), blood cell (ME1, BV173, MV411, NB4, REH, CEM-VBL, K562, HL60, HL60-ADR, supB15, 697), colon (HT-29, LoVo), and nervous tissue (NB5, NB17, PCL4199, PCL1643, PCL2021, BE2C). Mouse cDNA was obtained by the same procedure from the following 10 tissue types (Ambion^®^-Normal): brain, heart, thymus, lung, liver, embryo, kidney, spleen, ovary, and testicle; and four cell lines: adrenal (Y-1), fibroblast (primary culture of embryonic fibroblast cell), and blood cell (J774A, RAW264.7). Rat cDNA was also obtained by RT-PCR from the following 10 tissue types (Ambion^®^-Normal): brain, heart, thymus, lung, liver, embryo, kidney, spleen, ovary, and testicle. The *Taq *DNA polymerase kit (Roche^®^) was used with primers designed to target flanking exons of the AS exons to be verified. PCR products of expected sized were purified using the QIAquick Gel Extraction kit (QIAgen^®^) and auto-sequenced.

### Data sources

The original EST databases were generously provided by TIGR (The Institute for Genome Research [53]). The human, mouse, and rat EST databases used in this study were HGI (Human Gene Index) Release 15 with 524 Mb for 835,626 sequences, MGI (Mouse Gene Index) Release 14 with 433 Mb for 777,505 sequences, and RGI (Rat Gene Index) Release 13 with 101 Mb for 147,056. The original human, mouse, and rat genomic data were versions hg17 (or NCBI Human Build 35), mm5 (or NCBI Mouse Build 33), and rn3 (or NCBI Rat Build 2), respectively. These genomic sequences and the UCSC-annotated genes/transcripts (including human, mouse, and rat) were all downloaded from the UCSC genome browser [54]. The NCBI-annotated human, mouse, and rat genes/transcripts (RefSeq) were downloaded at [55]. The InterPro resource was downloaded at [50]. The ENACE programs and sequences of the ENACE-identified AS events are available at [56].

## Abbreviations

AS – Alternative Splicing

CDS – CoDing Sequence

UTR – UnTranslated Region

ENACE – Extracting Novel Alternative splicing variants from Cross-species EST resources

PSEP – Progressive Signal Extracting and Patching system

CRASA – Complexity Reduction Algorithm for Sequence Analysis

## Authors' contributions

FCC and TJC conceived the study. TJC developed the ENACE algorithm. CJC implemented the data analysis processes. JYH performed the experimental validation. FCC and TJC wrote the draft. All authors read and approved the final manuscript.

## Supplementary Material

Additional File 1 ENACE-identified cassette-on exons; The file includes related information of cassette-on exons identified by six ENACE processes. Click here for file

Additional File 2 ENACE-identified intron retentions; The file includes related information of intron retentions identified by six ENACE processes Click here for file

Additional File 3 ENACE-identified lineage-specific exons; The file contains the ENACE-identified exons specific to (1) human and mouse; (2) human and rat; and (3) mouse and rat. Click here for file

Additional File 4 Validation of the ENACE-identified cassette-on exons; The file contains experimental results and the used primer sets of the tested cassette-on exons identified by six ENACE processes. Click here for file

Additional File 5 Validation of the ENACE-identified intron retentions; The file contains experimental results and the used primer sets of the tested intron retentions identified by six ENACE processes. Click here for file

## Figures and Tables

**Table 1:** Extracting processes and identification results of ENACE

				Located in CDS			
ENACE processes	AS types	Meta-ENACE exons	Pass Rule 1	Pass Rule 2	Pass Rule 3	Located in UTR*	ENACE exons +
E_H-m_	Cassette-on	270	219	172	92	24	116
	Retained intron	38	-	12	5	3	8
E_M-h_	Cassette-on	315	260	208	98	22	120
	Retained intron	51	-	18	5	2	7
E_H-r_	Cassette-on	49	44	39	26	3	28
	Retained intron	7	-	3	1	1	2
E_R-h_	Cassette-on	328	244	194	85	2	87
	Retained intron	59	-	24	1	1	2
E_M-r_	Cassette-on	255	210	169	74	31	105
	Retained intron	202	-	65	14	2	16
E_R-m_	Cassette-on	2789	2017	1550	317	59	376
	Retained intron	679	-	220	40	17	57
Total		5042	2994	2674	758	167	925

.* Rules 2 and 3 only apply to prediction of coding exons.

**Table 2:** Properties of ENACE CDS exons (including 6 ENACE identifications).

**Cassette-on exons**	
No. of exons identified	692
Average length (bp)	86
Median length (bp)	63
Average number of supporting ESTs	1.3
Average sequence identity with the counterpart (%)	89
**Retained introns**	
No. of exons identified	66
Average length (bp)	766
Average length of retained intron (bp)	115
Average number of supporting ESTs	1.1
Average sequence identity with the counterpart (%)	86
No. of initial exons^1^	17
No. of terminal exons^2^	19
No. of single-exon transcripts^3^	6

The retained introns connect ^1 ^the first and the second (or ^2 ^the last and the second last) coding exons of the original transcripts. ^3 ^The retained introns connect the exons of originally two-exon transcripts to make single-exon transcripts.

**Table 3:** Experimental verification of ENACE CDS exons.

**Cassette-on exons**	Human vs. mouse		Human vs. rat		Mouse vs. rat	
	E_H-m_	E_M-h_	E_H-r_	E_R-h_	E_M-r_	E_R-m_
	
No. of ENACE CDS exons	92	98	26	85	74	317
No. of exons for experimental test	12	12	6	12	12	12
No. of verified exons (%)	10	10	3	8	9	7
	(83%)	(83%)	(50%)	(67%)	(75%)	(58%)
**Retained introns**	Human vs. Rodents				Mouse vs. rat	
	(E_H-m_+E_M-h_+E_H-r_+E_R-h_)				E_M-r_	E_R-m_
	
No. of ENACE CDS exons	12				14	40
No. of exons for experimental test	5				5	10
No. of verified exons	4				4	6
	(80%)				(80%)	(60%)
